# Association of OPA1 Polymorphisms with NTG and HTG: A Meta-Analysis

**DOI:** 10.1371/journal.pone.0042387

**Published:** 2012-08-03

**Authors:** Yatu Guo, Xia Chen, Hongtuan Zhang, Ningdong Li, Xiong Yang, Wenbo Cheng, Kanxing Zhao

**Affiliations:** 1 Tianjin Medical University, Tianjin, China; 2 Tianjin Eye Hospital, Tianjin Eye Institute, Tianjin Key Lab of Ophthalmology and Visual Science, Tianjin, China; 3 Second Hospital of Tianjin Medical University, Tianjin, China; 4 Krieger Mind/Brain Institute, Johns Hopkins University, Baltimore, Maryland, United States of America; 5 Department of Ophthalmology, the People’s Hospital of Changzhi, Shanxi, China; Purdue University, United States of America

## Abstract

**Background:**

Genetic polymorphisms of the Optic atrophy 1 gene have been implicated in altering the risk of primary open angle glaucoma (POAG), especially the susceptibility to normal tension glaucoma (NTG), but the results remain controversial.

**Methods:**

Multiple electronic databases (up to January 20, 2012) were searched independently by two investigators. A meta-analysis was performed on the association between Optic atrophy 1 polymorphisms (rs 166850 and rs 10451941) and normal tension glaucoma (NTG)/high tension glaucoma (HTG). Summary odds ratios (ORs) and 95% confidence intervals (CI) were estimated.

**Results:**

Seven studies of 713 cases and 964 controls for NTG and five studies of 1200 cases and 971 controls for HTG on IVS8+4C>T (rs 166850) and IVS8+32T>C (rs10451941) were identified. There were significant associations between the OPA1 rs10451941polymorphism and NTG susceptibility for all genetic models(C vs. T OR = 1.26, 95% CI 1.09–1.47, **p = 0.002**; CC vs. TT: OR = 1.52, 95% CI 1.04–2.20, **p = 0.029**; CC vs. CT+TT: OR = 1.64, 95% CI 1.16–2.33, **p = 0.005**; CC+CT vs. TT: OR = 1.21, 95% CI 1.02–1.44, **p = 0.032**). However, no evidence of associations was detected between the OPA1 IVS8+32C>T polymorphism and POAG susceptibility to HTG. Similarly, clear associations between the rs 166850 variant and NTG were observed in allelic and dominant models (T vs. C OR = 1.52, 95% CI 1.16–1.99, **p = 0.002**; TT+TC vs. CC OR = 1.50, 95% CI 1.13–2.01, **p = 0.006**) but not to HTG. In subgroup analyses by ethnicity, we detected an association between both OPA1 polymorphisms and risk for NTG in Caucasians but not in Asians. By contrast, no significant findings were noted between OPA1 variants for HTG, either in Caucasians or in Asians.

**Conclusions:**

Both the IVS8+4C>T and IVS8+32T>C variants may affect individual susceptibility to NTG. Moreover, stratified analyses for NTG detecting the effects of both OPA1 polymorphisms seemed to vary with ethnicity. Further investigations are needed to validate the association.

## Introduction

Glaucoma, the leading cause of irreversible blindness worldwide [1], is characterized by visual field defects, retinal ganglion cell death, and progressive degeneration of the optic nerve [1], [2], [3], [4]. Approximately half of all cases are of the angle closure type, which is prevalent among Asian populations [5], [6], [7], [8]. The remaining cases consist of primary open angle glaucoma (POAG) [9] and affect 70 million individuals worldwide [10], [11], [12]. POAG is clinically classified into high tension glaucoma (HTG), in which elevated intraocular pressure (IOP) is a major feature, and normal tension glaucoma (NTG), in which IOPs are consistently within the statistically normal population range [13], [14], [15], accounting for approximately a third of all POAG cases [13]. POAG is considered to be a multi-factorial disorder with a significant heritable component [16], [17], [18], [19], [20], [21], [22], [23], [24], [25]. Three causative genes have been identified thus far: optineurin (OPTN, OMIM 602432) on chromosome 10p14-15 [18], [26], myocilin (MYOC, OMIM 610652) on chromosome 1q24-25 [27]–[28], and WDR36 (OMIM 609669) on chromosome 5q21-22 [29], [30], but these account for fewer than 10% of patients with sporadic, adult-onset POAG. Multiple POAG susceptibility loci have been identified in populations from different ethnic backgrounds [31], [32], [33], [34], [35], [36], [37], [38], [39], [40], [41].The majority of the findings are conflicting, including those for the OPA1 gene located on chromosome 3.

The optic atrophy 1 (OPA1) gene [42] (OMIM 605290, chromosome 3q28) is a nuclear gene encoding a dynamin-related protein. As a family of GTPases, dynamins have been found to be ubiquitous in all human tissues tested and localized to mitochondria, with important functions in mitochondrial biogenesis and membrane integrity [43], [44]. Mutations in the OPA1 gene were at first considered to be reasonable candidates for autosomal dominant optic atrophy (ADOA) because the defective The OPA1 gene product may cause a derangement in mitochondrial metabolic function, including respiratory deficiency, which may be involved in the degeneration of retinal ganglion cells and atrophy of the optic nerve [45], [46], [47].The similarities between the clinical phenotypes and the finding that OPA1 is expressed in the optic nerve made OPA1 an excellent candidate susceptibility gene for POAG, or specifically for NTG [48], [49], [50].

In recent years, OPA1 polymorphisms have attracted widespread attention. Although several OPA1 polymorphisms have been investigated as risk factors for POAG, two polymorphisms, IVS8+4C>T and IVS8+32T>C, within the OPA1 gene have been the most extensively investigated so far. Aung et al. [48], [51] first reported the rs 166850 (IVS8+4C>T) polymorphism to be associated with NTG but not HTG. If so, such a finding would be beneficial for screening those people at risk of developing NTG. On the contrary, Mabuchi and his colleagues [52] reported that this polymorphism influences the phenotypic features in patients with HTG. Their findings, however, could not be replicated in all populations [53], [54], [55], [56]. Whether OPA1 gene polymorphisms may contribute to the pathogenesis of POAG is still vigorously debated.

To date, no meta-analysis has been conducted to validate the association of polymorphisms of OPA1 with normal tension glaucoma (NTG) and high tension glaucoma (HTG). Hence, we performed a meta-analysis of all eligible studies to derive a more precise estimation of the association, to help us better understand its possible influence on POAG.

## Methods

### Publication Search

MEDLINE, EMBASE, Science Citation Index, the Cochrane Library and the Chinese National Knowledge Infrastructure (CNKI) were searched up to the end of February 2012, using the key terms “OPA1 gene” or “optic atrophy 1 (autosomal dominant)” or “OPA1”, “polymorphism” or “SNP” or “single nucleotide polymorphism” or “variation” or “mutation”, and “glaucoma” or “primary open-angle glaucoma” or “POAG” or “high tension glaucoma” or “normal tension glaucoma” or “NTG” or “HTG”. Relevant publications were examined for references until no further studies were found.

### Inclusion Criteria

The following inclusion criteria were used to select literature for the meta-analysis: (1) case-control, nested case-control, or cohort studies; (2) description of the association of OPA1 polymorphisms with POAG; (3) the numbers of cases and controls and the number of different genotypes of IVS8+4C>T, IVS8+32T>C in the cases and controls were provided, or information that could help infer the results in the papers.

### Data Extraction

Two observers (YTG, XC) independently abstracted data from all eligible publications onto paper data collection forms. Two reviewers were blinded to the details (title, author and academic address) of these studies during assessment. Disagreements were resolved by discussion or consensus involving a third reviewer (HTZ) when required. The following items were collected from each study: first author’s surname, year of publication, statistical data, ethnicity, total number of cases and controls as well as numbers of cases and controls for each OPA1 genotype.

### Statistical Analysis

The Hardy–Weinberg equilibrium (HWE) in controls was recalculated in our meta-analysis. The chi-squared goodness of fit was used to test deviation from HWE (significant at the 0.05 level). The effect measure of choice was pooled OR with its corresponding 95% CI. The assumption of heterogeneity was checked with the Q-test. A P-value less than 0.10 for the Q test indicated a lack of heterogeneity among the studies. Based on the Q-test value, two models of meta-analysis were applied for dichotomous outcomes: A fixed-effects model, using the Mantel-Haenszel (M-H method), was used to calculate the pooled ORs when the Q-test value ≥0.1. By contrast, a random-effects (DerSimonian and Laird, D+L) model was utilized if the Q-test value < 0.1. First we compared allele frequencies (IVS8+4C>T T vs. C; IVS8+32T>C C vs. T) between cases and controls. We then examined OPA1 genotypes using additive (TT vs. CC; CC vs. TT), recessive (TT vs. CT+CC; CC vs. TT+TC) and dominant (TT+TC vs. CC; CC+TC vs. TT) genetic models for allele T and allele C. Furthermore, subgroup analyses were performed by ethnicity.

One-way sensitivity analyses were performed by iteratively removing one study at a time to assess the stability of the meta-analysis results. Cumulative meta-analysis was performed to evaluate the accumulation of evidence on the association between OPA1 polymorphisms and NTG/HTG. Finally, publication bias was qualitatively assessed by performing Begg’s funnel plots, and it was quantitatively evaluated by Egger’s test. P<0.05 was considered representative of statistically significant publication bias. All statistical analyses were performed using a commercial statistical softwore package(STATA statistical software Version 11.0; STATA Corporation, College Station, TX,US). Two-sided p-values <0.05 were considered statistically significant.

## Results

### Literature Search and Characteristics

**Figure 1:**
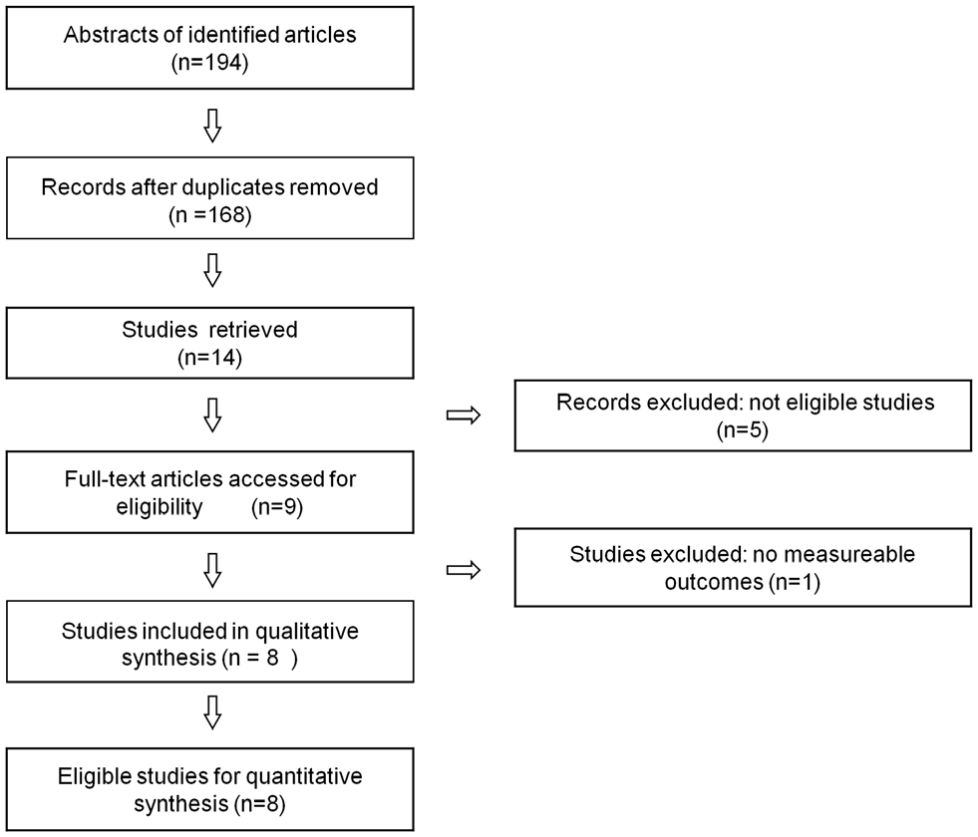
Flow chart of literature search and study selection.

The initial search yielded 194 articles. Based on the title, the content of the abstract and key words, 185 studies were excluded. Nine articles were reviewed in their entirety. One arm had to be excluded because of no data available [57]. Three studies for NTG [48], [53], [54], one study for HTG [56] and four studies for both NTG and HTG [52], [55], [58], [59] that met our inclusion criteria were included in this review. The flow chart of the literature search is shown in Figure 1. Seven studies [48], [52], [53], [54], [55], [58], [59] of 713 cases and 964 controls for NTG, and five studies [52], [55], [56], [58], [59] of 1200 cases and 971 controls for HTG were included in the meta-analysis of the OPA1 IVS8+4 C>T genotype. Similarly, seven studies (713 cases, 964 controls) for NTG and five studies (1200 cases, 971 controls) for HTG were included in the meta-analysis of the IVS8+32T>C genotype, of which, in one study [53], the distribution of the genotypes in the control group were not in Hardy-Weinberg equilibrium (HWE, Fisher’s exact test, P<0.05). Two independent cohort studies were reported in a paper by Aung and his colleagues [48]. For the meta-analysis of OPA1 polymorphisms for NTG, there were three studies on Caucasians [48], [53] [59], three studies on Asians [52], [54], [58], and one study on Barbadians [55]. Meanwhile, for the meta-analysis of OPA1 polymorphisms for HTG, two studies on Asians, one study on Caucasians, one study on Barbadians and one study of three different populations - Ghanaian, African American and Caucasian [56] - were included. Detailed study characteristics are summarized in Table 1.

**Table 1:** Main characteristics of studies included in this meta-analysis.

IVS8+4C>T(rs166850)					Cases			Control		
Author	Year	Ethnicity	Location	HWE	CC	CT	TT	CC	CT	TT
**HTG**										
Yao et al	2006	Barbados	US	0.99	47	1	0	46	2	0
Liu et al	2007	Caucasian	US	1.00	185	84	10	165	57	5
		Black		0.93	179	14	0	90	7	0
		Ghanaian		–	170	0	0	138	0	0
Mabuchi	2007	Asian	Japan	0.99	184	7	0	182	3	0
Fan et al	2010	Asian	HK, China	1.00	217	35	0	173	27	1
Yu-Wai-Man	2010	Caucasian	UK	0.17	49	16	2	59	13	3
**NTG**										
Aung et al^1^	2002	Caucasian	UK	0.75	57	26	0	86	14	0
Aung et al^2^	2002	Caucasian	UK	0.88	56	24	0	77	9	0
Powwell et al	2003	Caucasian	UK	0.73	41	16	4	111	53	4
Woo et al	2004	Asian	Korea	–	62	3	0	101	0	0
Yao et al	2006	Barbados	US	0.99	58	3	0	46	2	0
Mabuchi	2007	Asian	Japan	0.99	190	4	0	182	3	0
Fan et al	2010	Asian	HK, China	0.99	89	9	1	173	27	1
Yu-Wai-Man	2010	Caucasian	UK	0.17	41	26	3	59	13	3
**IVS8+32T>C(rs10451941)**					**Cases**			**Control**		
**Author**	**Year**	**Ethnicity**	**Location**	**HWE**	**TT**	**TC**	**CC**	**TT**	**TC**	**CC**
**HTG**										
Yao et al	2006	Barbados	US	0.66	20	16	12	16	26	6
Liu et al	2007	Caucasian	US	1.00	95	136	49	79	110	38
		Black		0.99	65	94	34	25	48	24
		Ghanaian		1.00	61	82	27	39	69	30
Mabuchi	2007	Asian	Japan	0.56	137	50	4	146	35	4
Fan et al	2010	Asian	HK, China	0.32	101	125	26	74	104	23
Yu-Wai-Man	2010	Caucasian	UK	1.00	12	36	19	21	37	17
**NTG**										
Aung et al^1^	2002	Caucasian	UK	0.27	47	36	0	72	28	0
Aung et al^2^	2002	Caucasian	UK	0.13	41	39	0	55	31	0
Powwell et al	2003	Caucasian	UK	**0.003**	17	27	17	43	104	21
Woo et al	2004	Asian	Korea	0.92	40	18	7	68	29	4
Yao et al	2006	Barbados	US	0.66	14	30	17	16	26	6
Mabuchi	2007	Asian	Japan	0.56	125	63	6	146	35	4
Fan et al	2010	Asian	HK, China	0.32	38	49	12	74	104	23
Yu-Wai-Man	2010	Caucasian	UK	1.00	11	37	22	21	37	17

HWE, Hardy-Weinberg equilibrium; HTG, high tension glaucoma; NTG, normal tension glaucoma; Black, African American; –, data not available.

### Meta-analysis Results

**Figure 2:**
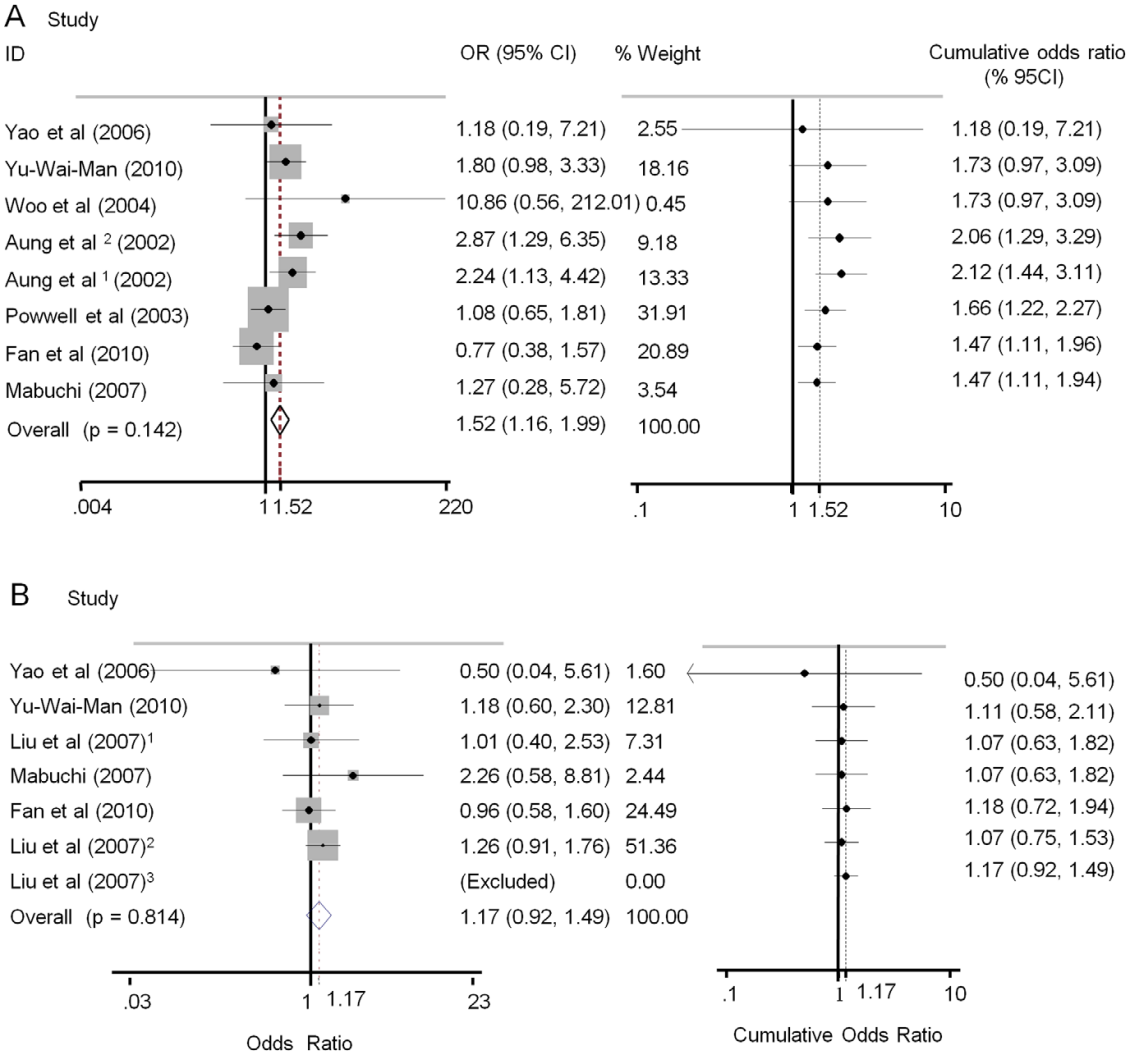
Forest plots describing overall meta-analysis (left) and cumulative meta-analysis (right) of the associations between IVS+4 T>C polymorphism and POAG risk. Odds ratios shown for individual studies for allelic model genotype contrasts (C vs. T). Cumulative odds ratios shown for each additional information step obtained by stepwise inclusion of every new study into pooled estimate. A. meta-analysis of association between IVS+4 T>C variant and NTG in order of sample size; B. meta-analysis of association between IVS+4 T>C variant and HTG in order of sample size.

**Figure 3:**
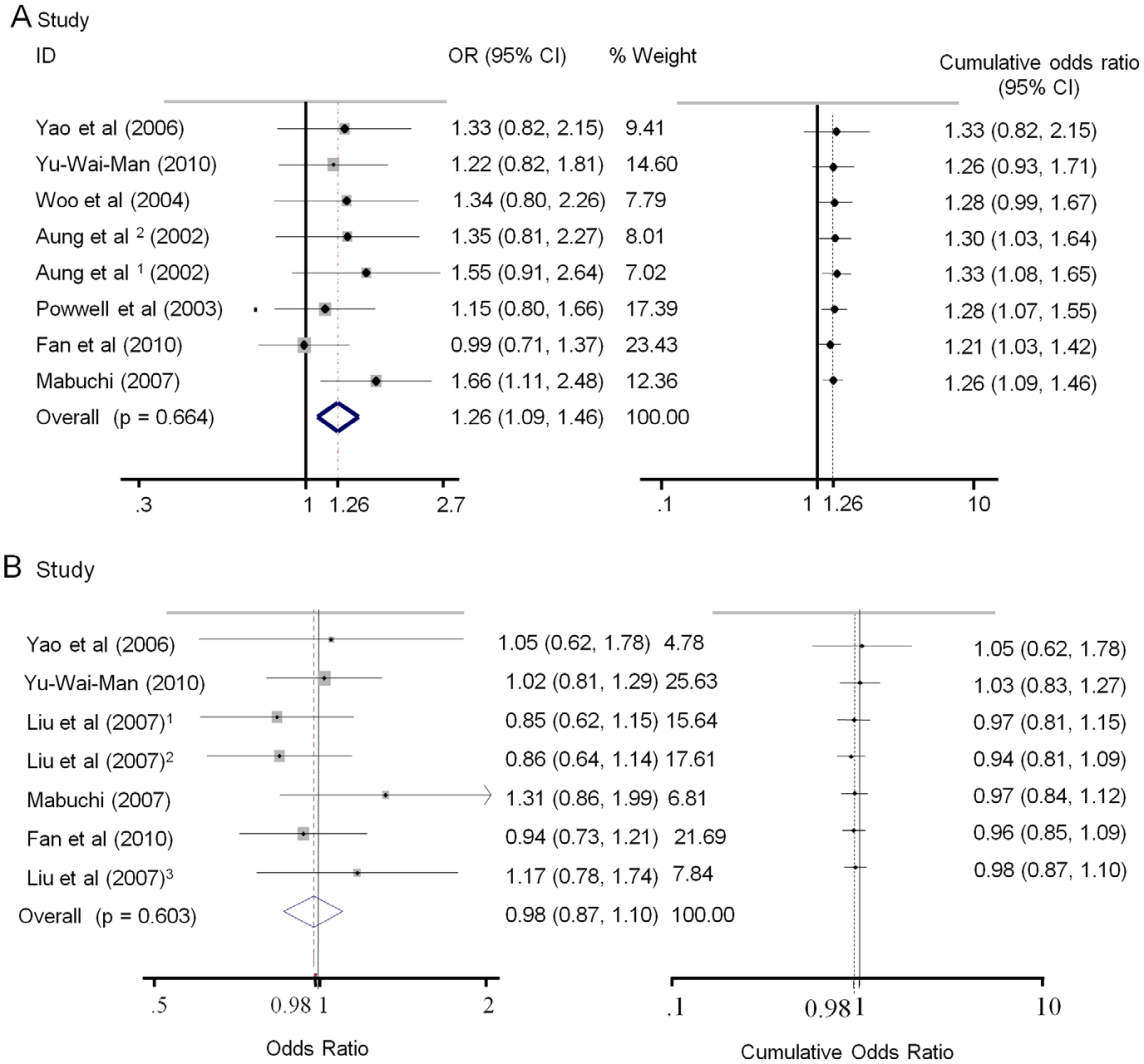
Forest plots describing overall meta-analysis (left) and cumulative meta-analysis (right) of the associations between IVS+32 C>T polymorphism and POAG risk. Odds ratios shown for individual studies for allelic model genotype contrasts (T vs. C). Cumulative odds ratios shown for each additional information step obtained by stepwise inclusion of every new study into pooled estimate. A. meta-analysis of association between IVS+32 C>T variant and NTG in order of sample size. B. meta-analysis of association between IVS+32 C>T variant and HTG in order of sample size.

The overall analyses suggested significant associations between the IVS8+32T>C polymorphism and normal tension glaucoma (NTG) susceptibility in all genetic models(C vs. T: OR = 1.26, 95% CI 1.09–1.47, P = 0.002; CC vs. TT: OR = 1.52, 95% CI 1.04–2.20, P = 0.029; CC vs. CT+TT: OR = 1.64, 95% CI 1.16–2.33, P = 0.005; CC+CT vs. TT: OR = 1.21, 95% CI 1.02–1.44, P = 0.032), and clear evidence of associations was found between the IVS8+4C>T variant and risk of NTG in allelic or dominant models (T vs. C OR = 1.52, 95% CI 1.16–1.99, P = 0.002; TT+TC vs. CC OR = 1.50, 95% CI 1.13–2.01, P = 0.006). However, no evidence of associations was detected between the two OPA1 polymorphisms and high tension glaucoma (HTG) susceptibility. Because a Q-test of heterogeneity among studies was nonsignificant in all genetic models, a fixed-effects model was used. (Data shown in Table 2 and Figures 2, 3).

**Table 2:** Results of meta-analysis for OPA1 polymorphisms and risk of primary open angle glaucoma.

Comparisons	Number of studies	OR	95%CI	P value	Heterogeneity		Effects model	Egger’s test
					I^2^	P value		P>|t|
**IVS8+4**								
**T vs C**								
**HTG**	5	1.17	0.92–1.49	0.20	0.00%	0.81	Fixed	0.74
Caucasian	2	1.25	0.93–1.68	0.15	0.00%	0.86	Fixed	
Asian	2	1.08	0.67–1.73	0.75	24.7%	0.25	Fixed	
**NTG**	7	1.52	1.16–1.99	**0.002**	35.9%	0.14	Fixed	0.64
Caucasian	3	1.70	1.25–2.31	**0.001**	42.8%	0.16	Fixed	
Asian	3	1.02	0.57–1.86	0.94	35.80%	0.21	Fixed	
**TT vs CC**								
**HTG**	5	1.23	0.52–2.91	0.65	0.00%	0.48	Fixed	–
Caucasian	2	1.43	0.57–3.59	0.45	0.00%	0.48	Fixed	
Asian	2	0.27	0.01–6.61	0.42	0.00%	0.48	Fixed	
**NTG**	7	1.95	0.71–5.34	0.19	0.00%	0.87	Fixed	0.83
Caucasian	3	1.96	0.66–5.75	0.22	0.00%	0.59	Fixed	
Asian	3	1.93	0.12–31.27	0.64	–	–	Fixed	
**TT vs TC+CC**								
**HTG**	5	1.15	0.49–2.71	0.75	0.00%	0.49	Fixed	–
Caucasian	2	1.33	0.53–3.32	0.54	0.00%	0.47	Fixed	
Asian	2	0.27	0.01–6.57	0.42	0.00%	0.49	Fixed	
**NTG**	7	1.82	0.67–4.94	0.24	0.00%	0.69	Fixed	0.90
Caucasian	3	1.79	0.61–5.22	0.29	0.00%	0.39	Fixed	
Asian	3	2.03	0.13–32.8	0.62	–	–	Fixed	
**TT+TC vs CC**								
**HTG**	5	1.17	0.90–1.52	0.23	0.00%	0.86	Fixed	0.85
Caucasian	2	1.24	0.89–1.72	0.20	0.00%	0.96	Fixed	
Asian	2	1.12	0.69–1.83	0.65	16.5%	0.27	Fixed	
**NTG**	7	1.50	1.13–2.01	**0.006**	35.9%	0.14	Fixed	0.90
Caucasian	3	1.70	1.22–2.38	**0.002**	48.3%	0.12	Fixed	
Asian	3	1.004	0.54–1.87	0.99	38.4%	0.20	Fixed	
**IVS8+32**								
**C vs. T**								
**HTG**	5	0.98	0.87–1.10	0.74	0.00%	0.6	Fixed	0.29
Caucasian	2	1.06	0.87–1.29	0.58	0.00%	0.58	Fixed	
Asian	2	1.03	0.83–1.28	0.80	41.8%	0.19	Fixed	
**NTG**	7	1.26	1.09–1.47	**0.002**	0.00%	0.56	Fixed	0.08
Caucasian	3	1.27	1.02–1.58	**0.031**	0.00%	0.67	Fixed	
Asian	3	1.24	0.99–1.56	0.06	50.00%	0.14	Fixed	
**CC vs. TT**								
**HTG**	5	0.92	0.71–1.19	0.54	0.00%	0.78	Fixed	0.42
Caucasian	2	1.11	0.75–1.72	0.56	0.00%	0.59	Fixed	
Asian	2	0.89	0.51–1.58	0.70	0.00%	0.79	Fixed	
**NTG**	7	1.52	1.04–2.20	**0.029**	0.00%	0.84	Fixed	0.1
Caucasian	3	1.51	0.87–2.61	0.14	0.00%	0.97	Fixed	
Asian	3	1.40	0.78–2.49	0.26	0.00%	0.42	Fixed	
**CC vs. TT+TC**								
**HTG**	5	0.94	0.74–1.20	0.64	0.00%	0.62	Fixed	0.35
Caucasian	2	1.10	0.75–1.63	0.62	0.00%	0.69	Fixed	
Asian	2	0.91	0.53–1.57	0.74	0.00%	0.93	Fixed	
**NTG**	7	1.64	1.16–2.33	**0.005**	0.00%	0.65	Fixed	0.51
Caucasian	3	1.75	1.06–2.89	**0.030**	0.00%	0.35	Fixed	
Asian	3	1.37	0.78–2.40	0.27	0.00%	0.45	Fixed	
**CC+TC vs. TT**								
**HTG**	5	0.99	0.86–1.14	0.9	0.00%	0.83	Fixed	0.63
Caucasian	2	1.05	0.82–1.33	0.72	0.00%	0.70	Fixed	
Asian	2	1.05	0.82–1.35	0.69	34.8%	0.22	Fixed	
**NTG**	7	1.21	1.02–1.44	**0.032**	0.00%	0.64	Fixed	0.42
Caucasian	3	1.20	0.93–1.55	0.16	0.00%	0.61	Fixed	
Asian	3	1.23	0.95–1.60	0.12	40.50%	0.19	Fixed	

**Figure 4:**
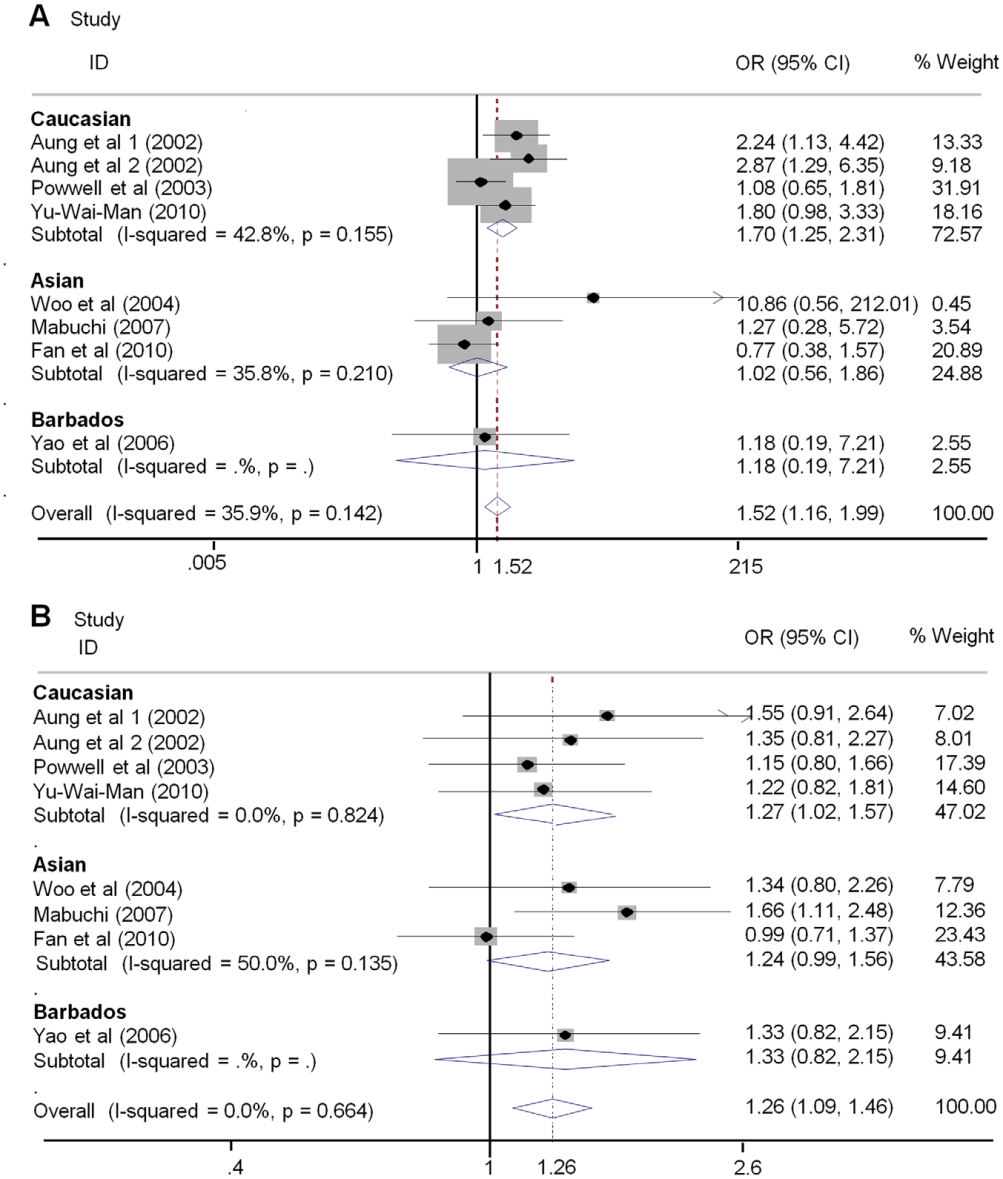
Forest plots describing subgroup analyses of the association between OPA1 polymorphisms and risk for NTG. The size of the square indicate the relative weight of each study.Bars,95% confidence interval(95% CI) A. subgroup analysis of IVS8+4 C>T stratified by ethnicity in order of publication year; B subgroup analysis of IVS8+32 T>C stratified by ethnicity in order of publication year.

**Figure 5:**
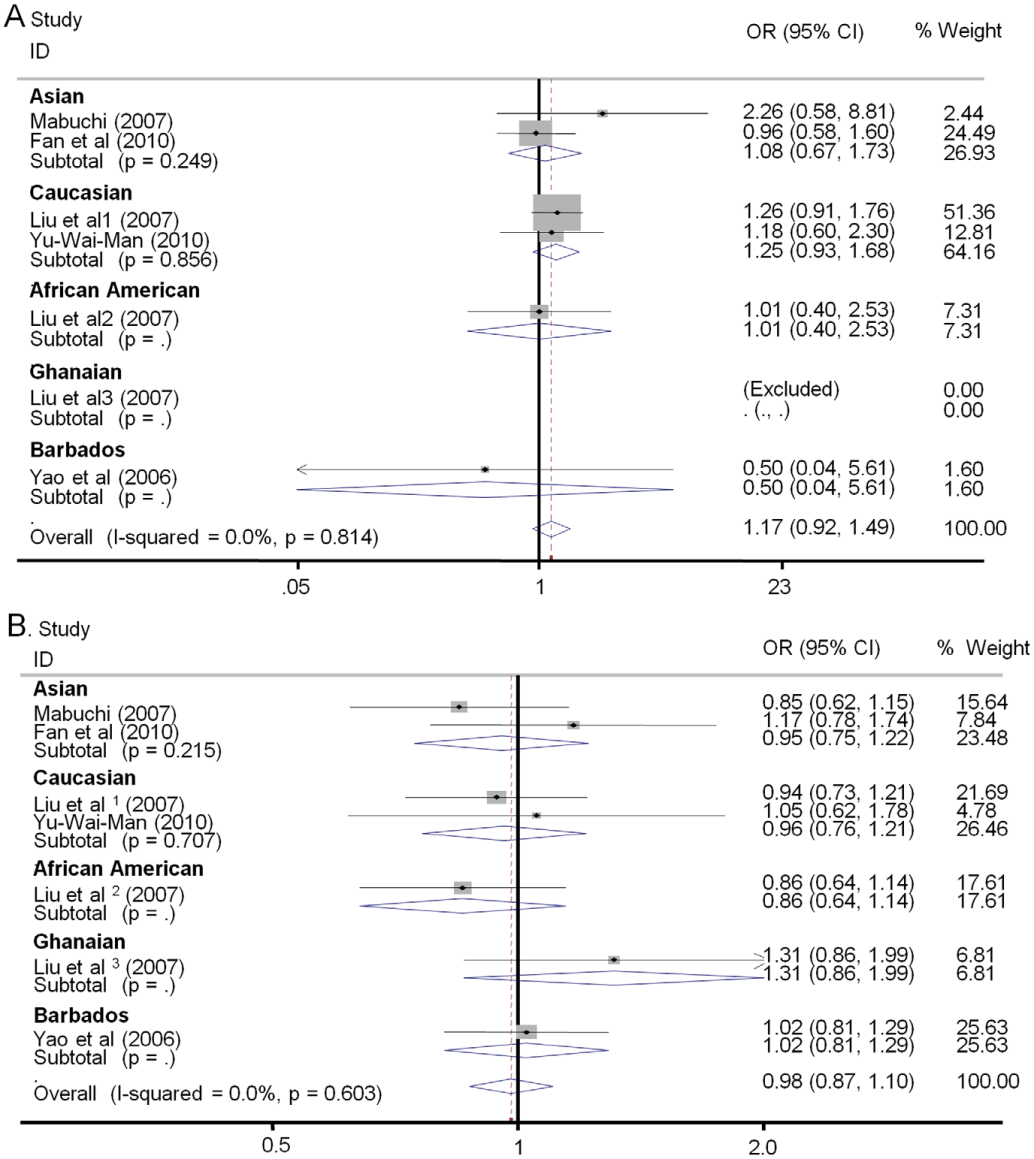
Forest plots describing subgroup analyses of the association between OPA1 polymorphisms and risk for HTG. The size of the square indicate the relative weight of each study.Bars,95% confidence interval(95% CI) A. subgroup analysis of IVS8+4 C>T stratified by ethnicity in order of publication year;B subgroup analysis of IVS8+32 T>C stratified by ethnicity in order of publication year.

To better understand the exact consequence of the relationship between OPA1 polymorphisms and normal tension glaucoma susceptibility, we investigated the effects of the OPA1 IVS8+4C>T and IVS8+32T>C genotypes on the occurrence of NTG by ethnicity. Overall, no evidence of association was observed in any genetic model between the OPA1 IVS8+4C>T or IVS8+32C>T variants and risk of normal tension glaucoma (NTG) in Asians. In contrast to the results for NTG risk in Asians, the analyses showed significant associations between both OPA1 polymorphisms and susceptibility to NTG in Caucasians (rs 166850 T vs. C OR = 1.70, 95% CI 1.25–2.31, P = 0.001; TT+TC vs. CC OR = 1.70, 95% CI 1.22–2.38, P = 0.002; rs 10451941 C vs. T OR = 1.27, 95% CI 1.02–1.58, P = 0.031; CC vs. TT+TC OR = 1.75, 95% CI 1.06–2.89, P = 0.03; Figure 4). In addition, stratified analyses by ethnicity were performed between the OPA1 polymorphisms and high tension glaucoma (HTG) risk. However, no significant finding was noted between either OPA1 polymorphism and risk of HTG, either in Asians or in Caucasians (Table 2 and Figure 5).

### Sensitivity Analysis and Cumulative Meta-analysis

To evaluate the robustness of the association results, a meta-analysis was performed repeatedly with each study removed. The results indicated that the fixed-effect estimates before or after the deletion of any single study were generally similar, suggesting a high stability of the meta-analysis results (data not shown). Cumulative meta-analysis of both OPA1 polymorphisms and NTG risk revealed that the summary ORs was larger than 1, and the 95% CI was reduced with accumulated sample size. By contrast, the pooled ORs of cumulative meta-analysis for HTG remained centered on 1 with increasing sample size, indicating that rs 166850 and rs 10451941 were unlikely risk variants for HTG, as shown in Figures 2 and 3.

### Publication Bias

**Figure 6:**
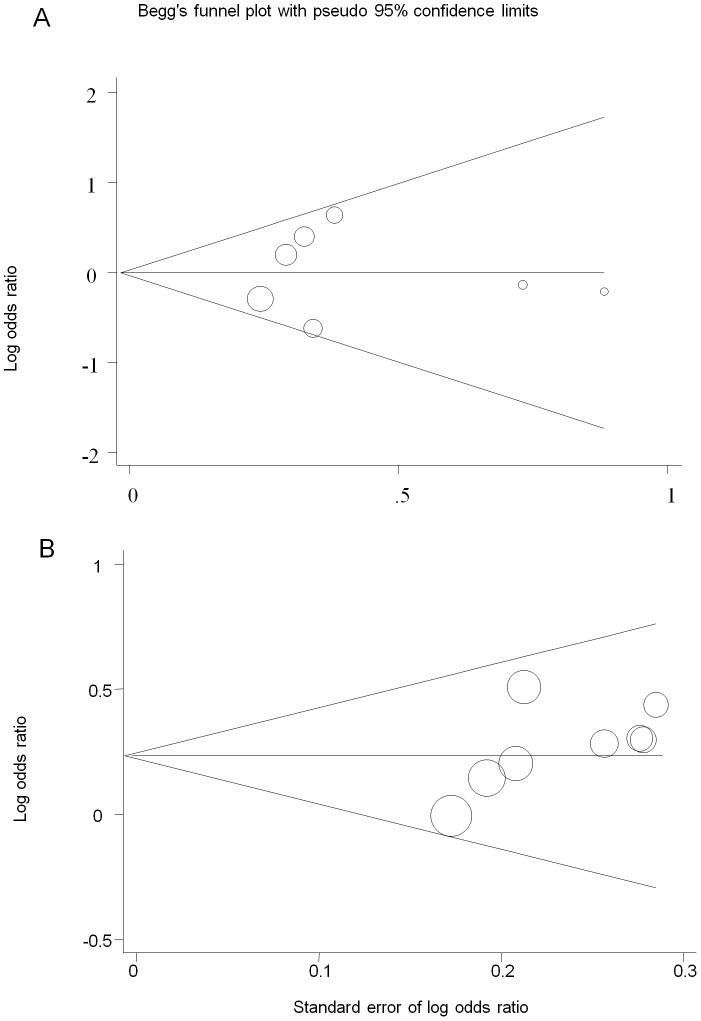
Begg’s funnel plot of OPA1 polymorphisms and NTG for allelic model. A: IVS8+4 T vs C; B:IVS8+32 C vs T. Each circle represents a separate study for the indicated association, and its size is proportional to the sample size of each study.

Publication bars were qualitatively assessed by Begg’s funnel plot and quantitatively assessed by Egger’s test. Neither Begg’s funnel plot nor Egger’s test detected obvious evidence of publication bias in the overall and subgroup analyses for all genetic models (Figure 6; data available in Table 2).

## Discussion

Primary open-angle glaucoma is considered to be a complex trait with a strong genetic component [12], [60]. OPA1, the gene responsible for autosomal dominant optic atrophy (ADOA) [61], represents a good candidate genetic risk factor for POAG [49], as the clinical phenotypes between ADOA and POAG are similar, and OPA1 is expressed in the retinal ganglion cells and optic nerve [62]. The OPA1 gene is related to mitochondrial biogenesis and respiration through its product of dynamin-related GTPase. The dysfunction of mitochondria caused by mutations in the OPA1 gene in ADOA is thought to be associated with apoptosis of retinal ganglion cells leading to optic neuropathy [50]. Since the identification of OPA1 polymorphisms, a number of studies have investigated the genetic effects of OPA1 polymorphisms on POAG susceptibility with conflicting results. Meta-analysis, as a powerful statistical method, can provide a quantitative approach to pooling variant results on the same topic to estimate and explain their diversity [63], [64]. This advantage stimulated us to conduct this meta-analysis of eight published case-control or cohort studies, which may help elucidate these phenomena and explore a more robust estimate of the effects of these polymorphisms on primary angle glaucoma.

In this meta-analysis, we found that the effect of the OPA1 polymorphisms on POAG risk may vary by subtype of POAG [49], [56] and ethnicity [54], [55], [56]. Studies by Aung et al. [48],Yao et al. [55] and Liu et al. [56] revealed that the association of OPA1 variants with POAG may be limited to patients with normal tension glaucoma, suggesting that a genetic mechanism favoring OPA1 would have a greater role in the pathophysiology of NTG than HTG. By contrast, Mabuchi and his colleagues [52] presented data that this polymorphism also influences the HTG phenotype and should therefore be considered a genetic risk factor not only for NTG but also for HTG in the Japanese population. Therefore, overall analyses were conducted on normal tension glaucoma (NTG) and high tension glaucoma (HTG). Strong evidence was found that the associations with NTG were significant for the IVS8+4C>T(rs 166850) and IVS8+32T>C (rs 10451941) polymorphisms. There was, however, no clear association between either OPA1 polymorphism and HTG, which is consistent with the findings of the largest cohort study, performed by Liu and his colleagues [56]. It is worth noting that the association between the presence of the C allele and the occurrence of HTG presented by Mabuchi is marginal [52]. The explanation for this discrepancy may be that the small sample size reduced its power to detect the association; alternatively, this discrepancy may result from different ethnicities. Thus, it is necessary to confirm the HTG risk in OPA1 variant carriers in other ethnic populations with large numbers of patients and well-defined diagnostic criteria. Meanwhile, reports on the associations of these two polymorphisms with NTG often conflict in different studies. Aung et al. [48] showed that the IVS8+4 C>T genotype was associated with NTG susceptibility, whereas Powell’s data [53] only supported an association of the IVS8+32T>C genotype with NTG, for which no independent association was seen by the Aung group. Our data showed that both the IVS8+4C>T and IVS8+32T>C polymorphisms are associated with risk of NTG. It should be noted that the distribution of the control group for IVS8+32T>C(rs 10451941) in Powell’s report [53] deviated from Hardy-Weinberg equilibrium, which may have been due to genotyping errors or selection bias in the control and/or population stratification. Therefore, as recommended by Attia [65],we conducted the meta-analysis again with this study removed. The results indicated that the estimates before or after deletion of the study were similar, suggesting a high stability of the meta-analysis results with little effect of this particular study.

As mentioned above, the results of many studies have represented that ethnic differences may affect genetic predisposition to NTG and HTG [52], [54]. For this reason, subgroup analyses on different ethnicities were performed. The results showed that OPA1 polymorphisms were significantly associated with NTG from studies of Caucasian individuals, but not in Asian individuals. In contrast to the results with NTG, no difference was found in the associations between OPA1 polymorphisms and HTG in Asians or Caucasians. The discrepancy may have arisen because studies with small sample sizes may be underpowered to detect a slight effect or may have generated a variable risk estimate. More large-scale cohort or case-control studies with sufficient power are needed to study HTG risk in carriers of OPA1 variants in different populations.

If the effect observed in this meta-analysis was truly without bias, several possibilities may explain why such an effect was detected in Caucasians but not in Asians. On the one hand, the possible role of ethnic differences in genetic background is suggested. On the other, the relevant environmental exposure in Asia may differ from that in Western countries. Furthermore, the genetic pathogenesis of NTG may be different between the two ethnic groups. Further studies involving different racial groups from the same geographic areas may help answer this question.

Some limitations in our study should be addressed, and the results should be interpreted with caution. First, controls were not uniformly defined. This study is a meta-analysis of case-control studies, most of which were hospital-based. Thus, some inevitable selection bias might exist in the results, and they may not be representative of the general population. Second, only published studies were included in this meta-analysis. Unpublished data, ongoing studies and articles published in languages other than English and Chinese were not sought, especially those with negative findings, which may have biased our results, although no obvious publication bias was apparent. Third, this meta-analysis was limited by the number of cases and controls as well as small sample sizes, especially in the subgroup analysis by ethnicity. Only a single study was conducted in Barbadian, Ghanaian, and African-American populations. Thus, additional studies are needed to evaluate the effect of these functional polymorphisms on POAG in different races. Fourth, our results were based on unadjusted estimates; a more precise analysis of the various groups should be conducted according to other factors, such as age and sex. Fifth, the genotyping methods used were different among these studies, which might have affected the results. This discrepancy between genotyping methods highlights the need for implementing rigorous quality control procedures in future studies. Finally, the existing literature lacks information about potential gene-gene or gene-environment interactions. Given that the role of several environmental factors in the pathogenesis of open angle glaucoma is established, further research should be performed in this direction.

In conclusion, the results of this meta-analysis suggest that two OPA1 polymorphisms [IVS8+4C>T (rs166850) and IVS8+32T>C (rs10451941)] are associated with an increased risk for normal tension glaucoma. It is also worthwhile to note that OPA1 polymorphisms may be involved in the pathogenesis of NTG in Caucasians but not in Asians. Due to the limitations discussed above, well-designed large-scale cohorts or case-control studies are warranted to confirm our findings.
